# Importance of Nanoparticles for the Delivery of Antiparkinsonian Drugs

**DOI:** 10.3390/pharmaceutics13040508

**Published:** 2021-04-08

**Authors:** Sara Silva, António J. Almeida, Nuno Vale

**Affiliations:** 1 OncoPharma Research Group, Center for Health Technology and Services Research (CINTESIS), 4200-450 Porto, Portugal; saracpsilva21@gmail.com; 2 Faculty of Pharmacy, University of Porto, Rua de Jorge Viterbo Ferreira 228, 4050-313 Porto, Portugal; 3 Research Institute for Medicines (iMed.ULisboa), Faculdade de Farmácia, Universidade de Lisboa, 1649-003 Lisbon, Portugal; aalmeida@ff.ulisboa.pt; 4 Faculty of Medicine, University of Porto, Al. Hernâni Monteiro, 4200-319 Porto, Portugal

**Keywords:** Parkinson’s disease, nanoparticles, drug, treatment, delivery systems, administration routes, nanotheranostics

## Abstract

Parkinson’s disease (PD) affects around ten million people worldwide and is considered the second most prevalent neurodegenerative disease after Alzheimer’s disease. In addition, there is a higher risk incidence in the elderly population. The main PD hallmarks include the loss of dopaminergic neurons and the development of Lewy bodies. Unfortunately, motor symptoms only start to appear when around 50–70% of dopaminergic neurons have already been lost. This particularly poses a huge challenge for early diagnosis and therapeutic effectiveness. Actually, pharmaceutical therapy is able to relief motor symptoms, but as the disease progresses motor complications and severe side-effects start to appear. In this review, we explore the research conducted so far in order to repurpose drugs for PD with the use of nanodelivery systems, alternative administration routes, and nanotheranostics. Overall, studies have demonstrated great potential for these nanosystems to target the brain, improve drug pharmacokinetic profile, and decrease side-effects.

## 1. Introduction of Parkinson’s Disease

Over the years with increases of life expectancy, neurodegenerative diseases have become common causes of mortality and morbidity worldwide, affecting more the elderly population. Neurodegenerative diseases are characterized by a heterogenous clinical presentation, and successful diagnosis is crucial to ensure good treatment [1]. For instance, Parkinson’s disease (PD) is the second most prevalent neurodegenerative disease after Alzheimer’s disease. It is estimated that PD affects around ten million people worldwide, with an incidence of 3% of the population over 80 years old [2,3,4].

In this review, we give a brief overview of PD, its risks factors, and its current treatment, with a focus on alternative drug delivery routes and systems that have been explored so far for drug repurposing and theranostics tools.

PD was first described by James Parkinson in 1817 [3]. Clinical manifestations of PD are characterized by motor and non-motor symptoms (cognitive changes, behavior/neuropsychiatric changes, and autonomic dysfunctions). Motor symptoms includes resting tremors, rigidity, postural instability, and bradykinesia (the inability of PD patients to do fine motor tasks). Non-motor symptoms comprise sleep disturbances, anosmia (loss of sense of smell), constipation, and speech changes. In addition, around 60% of PD patients develop dementia, and around 50% are affected by depression [2,5,6].

**Figure 1:**
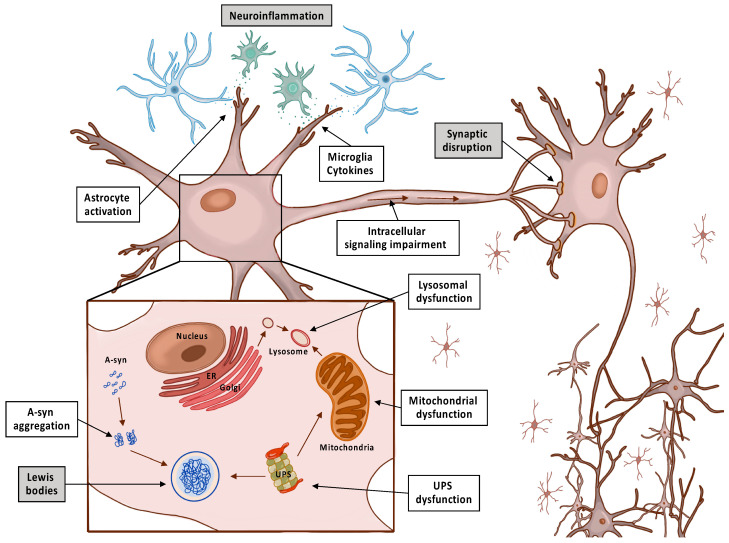
Schematic representation of the major hallmark events leading to Parkinson’s disease with the formation of Lewis bodies, ubiquitin proteosome system (UPS) dysfunction, neuroinflammation, and synaptic disruption with neurodegeneration. Endoplasmic reticulum (ER); alfa-synuclein (A-syn).

In the early stages of PD, it is usually a challenge to determine an accurate diagnose due to the variety of parkinsonism forms that exist with similar symptoms of PD. Some examples include infectious disease (encephalitis, prion disease, neurosyphilis, and toxoplasmosis), drug-induced parkinsonism, Lewy bodies disease, progressive supranuclear palsy, corticobasal degeneration, and multiple system atrophy [7]. In clinical practice, PD is diagnosed from an evaluation of a patient’s symptoms, genetic tests, a drug challenge test with levodopa (dopaminergic response), autonomic function testing, olfactory testing or neuroimaging (magnetic resonance imaging (MRI), positron emission tomography (PET)) and midbrain sonography) [7]. After diagnosis, expected life span of PD is around 6.9–14.3 years, the main cause of death is not directly linked to the disease, and the progression of the disease varies in each individual [8]. The pathology of PD is characterized by a loss of dopaminergic neurons within the substantia nigra pars compacta and the development of lewy bodies that are eosinophilic cytoplasmatic inclusions composed of aggregated proteins, mainly α-synuclein (α-syn) [9]. These α-syn aggregates interfere with microtubule-based subcellular transport, thus contributing to synaptic dysfunction and the destabilization of neuronal homeostasis. Moreover, several studies had demonstrated that these toxic α-syn aggregates are able to propagate throughout the brain, similar to prion virus disease. Even though these PD hallmarks are present in post-mortem PD patient autopsies, the primary cause of the early stage of the disease is still unclear [10]. Several studies conducted so far have proposed multiple pathogenic and epigenetic mechanisms that must underlie the development of the disease, such as mitochondrial dysfunction, lysosomal and proteasome dysfunction, neuroinflammation (microglia and astrocyte impairment), and oxidative stress [11,12,13,14]. All of these correlated pathologic events lead to imbalance of brain homeostasis, dopamine depletion, synaptic dysfunction, and neuronal loss [15], as shown in Figure 1.

Several risks factors could potentiate the development of PD such as age, genetic factors, environmental factors, and lifestyle behaviors (reviewed in more depth in [16]). The majority of PD patients have an idiopathic disease, around 15% have a family history, and 5–10% present a monogenic form of the disease. Recent research collected at least 23 loci and 19 disease-causing genes for PD, including 10 autosomal-dominant genes and 9 autosomal-recessive genes [17]. Depending on the type of gene mutation, the pathophysiological hallmarks and clinical manifestations are different. Some gene mutations are directly linked with the disruption of α-syn function and the accumulation of, e.g., PARK 1, PARK 4, and LRRK2 are directly linked with disruption of α-syn function and accumulation [18]. In addition, gene mutations such as PINK 1, PARKIN, UCHL 1 are linked with malfunction of the ubiquitin–proteasome system and the autophagy system. These systems enables the efficient clear of misfolded proteins and remotion of disrupted mitochondria in neurons. Dysfunction of these regulatory mechanisms facilitated accumulation of misfolded proteins such as α-syn and consequently leads to the formation of lewy bodies. Moreover, the impairment of lysosomal function has been linked with PD and alterations to the ATP13 A2 gene [14]. Several environmental factors have been related as well with increased risk of developing PD such as exposure to certain toxins, insecticides (paraquat and rotenone), narcotic drugs, and some solvents [16].

## 2. Considerations about PD Treatment

**Figure 2:**
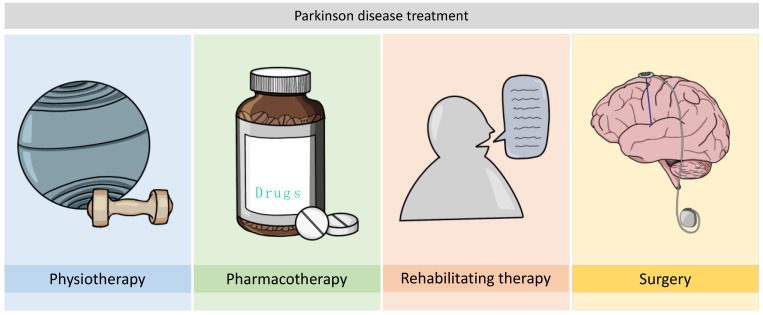
The current Parkinson’s disease treatment introduced to recently diagnosed patients.

The treatment of PD starts when motor signs are developed. Current treatments include pharmacologic therapy, physical therapy, rehabilitating therapy and surgery (Figure 2). Physical therapy and exercise are beneficial in PD patients for both motor and non-motor symptoms. The activities include speech therapy, nutrition, physiotherapy, and support groups.

**Figure 3:**
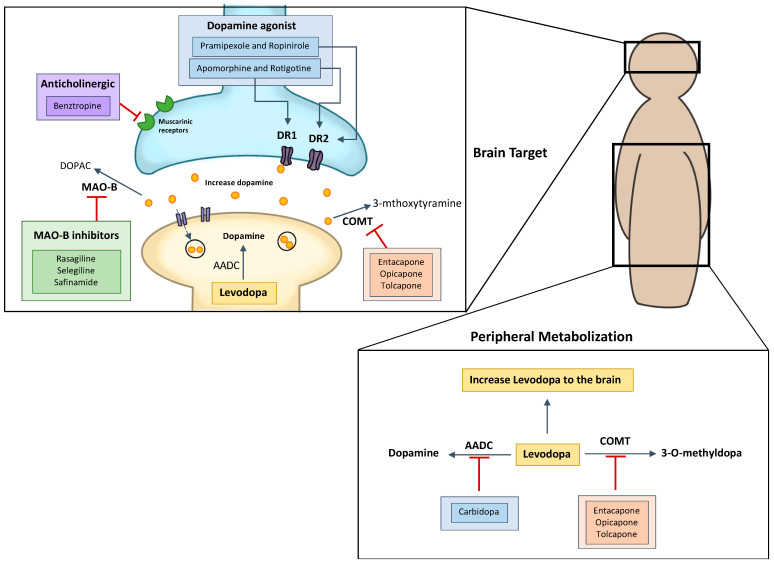
Parkinson’s disease (PD) pharmacotherapy mechanism and sites of action. COMT: Catechol *o*-methyltransferase; MAO-B: mono-amide oxidase B. Aromatic L-amino acid decarboxylase (AADC); Catechol *o*-methyltransferase (COMT); mono-amide oxidase B (MAO-B); Dopamine receptors (DR); dihydroxyphenyl acetic acid (DOPAC).

The current pharmacologic therapy is based on the symptomatic relief of motor and non-motor symptoms (just reported in [8]). Today, the main strategy for motor symptom treatments is based on an increase of dopamine levels, mimic dopamine effect or decrease dopamine metabolization and storage. There are seven types of drugs that clinicians use to treat PD: levodopa/carbidopa, dopamine agonists, mono-amide oxidase B (MAO-B) inhibitors, injectable dopamine agonists, *N*-methyl-D-aspartate receptor (NMDA) inhibitors, and anticholinergic drugs (Figure 3) [8]. For initial therapy, levodopa, non-ergot dopamine agonists, and MAO-B inhibitors can be used. Even though levodopa is the most effective treatment so far, early use is associated with the early development of dyskinesia (involuntary muscles movements). Levodopa alternative drugs can be used for PD treatment but with the progression of the disease the therapy starts to fail, and motor dysfunction starts to develop. Eventually, levodopa has to be included in the treatment in order to ensure adequate motor symptom relief [19]. Moreover, there are some clinicians that start levodopa treatment at the beginning and only use adjunctive and combination therapy when motor fluctuations (‘’on-time” and “off-time”) and dyskinesia appear. All of these adjunctive compounds are able to improve the pharmacokinetics of levodopa [20] (Figure 3).

### 2.1. Levodopa and Carbidopa

Levodopa is a prodrug of dopamine that was discovered in 1961 and is considered a replacement treatment of dopamine [19]. Levodopa facilitates central nervous system (CNS) penetration and brain dopamine delivery. Levodopa is still the most potent pharmacologic compound for treating PD [19]. Although dopamine is a great potent compound, it presents a low oral bioavailability, and only 1% of levodopa reaches the brain. In addition, the peripheral release of dopamine negatively affects different peripheral body functions such as decrease intestinal motility, insulin production, and vasodilation. So, co-administration with carbidopa is crucial to decrease the peripheral breakdown of levodopa and avoid side-effects. Carbidopa is an inhibitor of aromatic L-amino acid decarboxylase [21]. These compounds do not pass blood–brain barrier (BBB) which contributes to a higher therapeutic efficacy of levodopa in the brain and decrease peripheral side-effects [22,23,24]. Still, chronic long-term treatment cause motor complications such as dyskinesia.

### 2.2. Dopamine Agonists

#### 2.2.1. Apomorphine

Apomorphine is used in PD due the capacity to activate D1 like and D2 like receptors and its lipophilicity proprieties enables crossing the BBB. Unfortunately, apomorphine presents a short half-life (33 min), which necessitates administration through subcutaneous injections several times a day. The administration of this therapy is usually applied in the advance stage of PD to reduce off-time and dimmish motor fluctuations from levodopa treatment [25,26,27].

#### 2.2.2. Pramipexole

In 1997, pramipexole—a novel non-ergolinic dopamine agonist—was approved to treat PD. Pramipexole is an aminobenzothiazole with the full stimulation of the D2 subfamily dopamine receptor. After oral administration, it can reach 90% bioavailability and have protein binding of less than 15%, and an increase half-life (8–12 h) however can cause severe side-effects such as nausea, vomiting, hypotension, impulsive control disorder, and hallucinations [28,29,30].

#### 2.2.3. Ropinirole

Ropinirole is a non-ergoline selective dopamine D2 agonist, and it can be administered through all stages of PD. The beneficial outcomes of this therapy in the early stages of PD include a delay of the development of dyskinesias and a great efficacy to decrease motor symptoms and improve non-motor symptoms (sleep disturbances). In addition, this compound is administered through a silicone-based transdermal patch for a period of 24 h, at which point the systemic circulation results in a low protein binding and good half-life. Still, some adverse effects such as nausea, dizziness, somnolence, headache, vomiting, fatigue, and pain can occur [31,32].

#### 2.2.4. Rotigotine

Another option for PD treatment is the use of rotigotine, which is a non-ergolinic dopamine agonist of dopamine receptor families D3, D2, and D1 that uses a transdermal system. This compound can be used as monotherapy in early stage of PD and adjunctive therapy with levodopa in advanced PD. Some adverse effects are consistent with the over-stimulation of peripheral dopamine receptors, and they include nausea, somnolence, and application site reactions [31,33,34].

### 2.3. MAO-B Inhibitors

Another current strategy chosen to treat PD is the use of MAO-B inhibitors. MAO-B is an enzyme responsible for dopamine degradation. Thus, by inhibiting dopamine degradation, the levels of dopamine increase. Some examples of MAO-B inhibitors are rasagiline and selegiline. Even though MAO-B inhibitors present less effects, they also have less side-effects compared to treatments. Furthermore, several studies have demonstrated that another possible key feature of these compounds is their ability to act as neuroprotectants from oxidative stress [35,36].

### 2.4. Catechol O-Methyltransferase Inhibitors

Catechol *o*-methyltransferase (COMT) is responsible for the conversion of different catechols (like dopamine and epinephrine) and is another PD target therapy with the inhibition of this dopamine metabolization. Levodopa can be metabolized by decarboxylase in the peripheral system, and the co-administration of carbidopa will inhibit this process; however, once the system is occupied, levodopa is also metabolized by COMT in the periphery. Thus, the use of COMT inhibitors can prevent peripheral metabolization and allow for more levodopa to reach the brain. For instance, COMT inhibitors can extend the half-life of levodopa by 85% and increase absolute bioavailability. Some examples of these compounds include entacapone and tolcapone (oral formulation). Both of these compounds have been used as adjunctive therapy to control motor fluctuations acquired by patients from chronic levodopa treatment [37,38,39].

### 2.5. Anticholinergic

#### 2.5.1. Trihexyphenidyl

Trihexyphenidyl is an oral anticholinergic agent used to treat motor symptoms of PD and movement disorders. Trihexyphenidyl blocks central cholinergic receptors allowing maintenance of cholinergic transmission in the basal ganglia inhibiting the reuptake and storage of dopamine. This compound is usually used as adjunctive therapy with levodopa in early stage of PD. Trihexyphenidyl is available in tablets form (2 mg and 5 mg) and elixir form. The side-effects are nervousness, confusion, drowsiness, tachycardia, constipation and nausea [40].

#### 2.5.2. Benztropine

Another anticholinergic agent used in PD treatment as adjunctive therapy is benztropine. Benztropine belongs to muscarinic receptor antagonists’ class. Mechanisms of action includes reduction of the central cholinergic effect with the inhibition of muscarinic receptors (that inhibit the reuptake and storage of dopamine). This compound is not chosen often due to its several contraindications [41].

### 2.6. Amantadine

Amantadine is a non-competitive NMDA-receptor antagonist and was first used in 1960 as antiviral drug but soon showed a reduction of dyskinesias in PD patients. Thus, researchers found that an imbalance of dopamine levels is associated with an increased concentration of extracellular glutamate, which increase the expression and activity of NMDA-type glutamate receptors. By acting on this mechanism, amantadine is able to control glutamate concentrations and reduce the motor fluctuations present in PD. Still, this compound response is different between patients, and even though it has a half-life of 17 h, a two- or three-times daily dose is required [42,43].

### 2.7. Neurosurgical Treatments

The surgical approach is used when pharmaceutical therapy is no more beneficial to the patient, as in cases of severe motor fluctuations, dyskinesia, hallucinations, and intractable tremors. Deep brain stimulation is usually conducted at an advanced stage of PD and is characterized by the insertion of electrodes into the target area of the brain. After levodopa treatment, surgical intervention is the second most effective therapeutic showing benefits for up to five years. Although some disadvantages have to be taken in account when performing this type of treatment. Neurosurgical treatment do not improve speech or swallow issues, can have complications (infection, stroke, and seizures) and some patients reported cognitive decline [44,45].

## 3. Nanoparticles as Drug Delivery System Repurposing in PD

Though current PD therapy with dopamine replacement is effective in reducing motor symptoms, the long-term drug treatment and progression of PD pathology consequently leads to a loss of drug effectiveness and an increase of side-effects [46]. In addition and as mentioned above, some of the available drugs used to treat PD present a poor pharmacokinetic profile, low diffusion through the BBB, and limited bioavailability. Therefore, there is a need to develop new strategies that are able to extend and control the release of drugs. Recent advances in nanotechnology demonstrated that nanoparticles (NPs) are able to facilitate effective drug delivery, improve bioavailability, decrease pharmacokinetic side-effects, decrease drug dosage, and increase target delivery to the brain against a wide range of diseases [47]. NPs are versatile, nanosized colloidal systems and can be biodegradable or non-biodegradable depending on the nature of the polymer or material used. There have been a wide variety of nanosystems developed so far, and they can be divided into three classed: organic (liposomes, lipid, protein, or polymers), hybrid (nanofoams), and inorganic (metal or salt) [48]. Depending on the type of material chosen, there are different ways to produce them and their final desired function or activity. For instance, the use of inorganic materials to produce nanosystems—metals or quantum dots, in particular—is usually applied to develop delivery imaging agents [49]. On the other hand, the use of organic materials is linked to a higher biocompatibility and a low material toxicity [50]. So, when developing NPs, it is critical to understand and determine their biological fate, toxicity, drug release capability, and stability. For example, NPs with a particle size of less than 5 nm are rapidly cleared by the renal system, NPs bigger than 200 nm accumulate in the spleen due to interendothelial cell slits, and NPs between 2–5 µm in size tend to accumulate in the capillaries [51]. Other important physicochemical parameters that should be analyzed are surface properties such as hydrophobicity and zeta potential. The hydrophobicity analysis of an NP can determine its in vivo fate and the opsonization in its surface. On the other hand, zeta potential analysis also determines in vivo fate and stability. Usually, NPs with negative and neutral surface charges tend to have longer circulation times with less protein absorption, but NPs with a positive surface charge have an increase intracellular permeability in tumor sites. Moreover, a zeta potential above ±30 mV is linked to a higher NP stability in suspension and less aggregation [51]. In addition to this, nanoparticles can be functionalized and optimized with different molecules in order to confer targeting abilities and decrease systemic opsonization. NPs can be functionalized with polyethylene glycol (PEG), poloxamer, polysorbate 80 (T80), peptides, antibodies, transferrins, and more [52].

An increasing number of studies have demonstrated that nanoparticles can enhance drug delivery through both conventional and unconventional administration routes, which is crucial to explore alternatives in progressively debilitating diseases such as PD. In the next section, we review some already-developed nanosystems to deliver PD-available drugs through different modes of administration that could be applied in future therapeutic approaches.

### 3.1. Parenteral/Intravenous Delivery

Parenteral delivery is the most suitable route for drugs that have a low gastrointestinal track absorption. The main advantages of this route are the almost instantaneous onset of action, avoidance of first-pass metabolism, and enabling of complete drug bioavailability even at low doses. This mode of administration allows NPs to have direct systemic exposure and improve drug delivery to the brain. Different types of NPs have been prepared for the efficient delivery of PD drugs through parenteral administration [53,54]. For instance, Hwang and his team developed perfluoropentane nanobubbles encapsulated with apomorphine in order to increase systemic stability and ensure BBB passage. The particularly of this work is that the authors used a specific type of nanobubble and combined it with ultrasound pulses to temporarily disrupt the BBB and allow for apomorphine passage. The nanobubble optimization process showed a particle size of around 150–380 nm. The increase size between formulations was related to an increased oil and fluorocarbon ratio. In addition, the authors stated that the presence of cholesterol in these types of formulations was crucial to guarantee a high stability. The authors conducted plasma stability studies and demonstrated that apomorphine integrity and sustained release were obtained in the systems with higher oil and perfluoropentane percentages. Furthermore, plasma release studies demonstrated that apomorphine release from nanobubble system was higher when ultrasounds were applied [55].

Barcia and his co-workers developed a biodegradable polymeric NP loaded with ropinirole (RP-loaded poly lactic-co-glycolic acid (PLGA)) and evaluated its therapeutic effect in a rotenone-induced animal model of PD. The optimized formulation presented a size of less than 200 nm, a maximum encapsulation efficiency (EE) of 74.8%, and a zeta potential of −14.25 mV. The in vivo therapeutic effect showed that the intravenous administration of RP-loaded PLGA decreased neurodegeneration and reduced astrogliosis [56]. In another study, Somasundoram and his team developed a chitosan nanosuspension to co-deliver the PD drugs pramipexole and hesperidin (an antioxidant). The NPs had a particle size of 188 nm, a zeta potential +46.7 mV, and an EE of 72.3%. In addition, the authors demonstrated that the dual delivery system successful delivered the drugs into the brains of mice, and a histopathologic evaluation showed the reversed degeneration of neurons in rotenone-induced mice [57]. Furthermore, chitosan NPs were developed by Ray and his team to efficiently encapsulate ropinirole and target the brain. The chitosan NPs were prepared by ionic gelation crosslinked with T80. A laser scattering analysis demonstrated a particle size of 233 nm and a zeta potential of −19.6 mV. In vivo biodistribution studies demonstrated an increased accumulation in the brain after 1 h of administration when compared to ropinirole solutions of 4780 and 1621 ng/mL. Moreover, these results demonstrated that the T80 coating led to an enhanced brain accumulation when compared to non-coated chitosan NPs [58]. Javan and his-team developed a long-acting formula of pramipexole encapsulated in polymeric poly-(3-hydroxybutyrate-co-3-hydroxyvalerate) (PHBV)-based NPs. The physiochemical analysis showed a particle size of 227–642 nm, an EE of 39.6–87.2%, and steady release of 5.5% in 24 h and 89.9% within 30 days [59]. In another work, graphene oxide nanohybrids presented high surface areas, two-dimensional structures, high biocompatibility, and wide variety of applications (gene delivery, biosensor, medical imaging, tissue engineering, and more). In this particular case, those nanohybrids were able to incorporate trihexyphenidyl. Jawanjal and his team used this type of nanoparticle and developed a graphene oxide system to deliver trihexyphenidyl [60].

**Figure 4:**
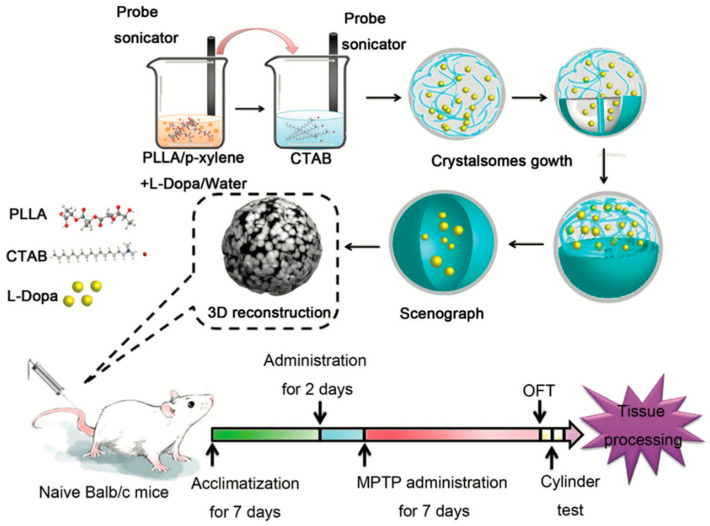
LD crystalsome preparation and experimental dosing The LD crystalsomes were obtained after quenching to a pre-set crystallization temperature for 48 h. The synthesized LD crystalsomes were given to the naive mice twice. Then all mice were administrated with MPTP for 7 days to the induced Parkinson-like mouse model. Mice were sacrificed after the behavioral test and examined for pathological changes. (Reproduced with permission from [65], Royal Society of Chemistry, 2013). 1-methyl-4-phenyl-1,2,3,6-tetra-hydropyridine (MTPT); Poly (L-lactic acid) (PLLA).

Levodopa chronic administration in long-term therapy can lead to the development of levodopa-induced dyskinesia and negatively affect the quality of life in patients. In the work by Zhou and his-team, PLGA NPs loaded with levodopa were developed via the double emulsion solvent evaporation technique. The authors observed that increased PLGA concentrations led to an enhanced organic phase viscosity, resulting in increased nanoparticle sizes due to higher resistance to the droplets breaking down. On the other hand, an increase of the poly (vinyl alcohol) (PVA) concentration led to a decrease of particle size, thus promoting emulsion stability. The EE of levodopa varied from 32.1 ± 2.23% to 62.2 ± 1.61%, depending on polymer and emulsifier concentrations [61]. In another work, the authors developed manganese oxide-based NPs that were functionalized with levodopa for use as a new magnetic resonance imaging (MRI) contrast agent and drug delivery system. The results demonstrated a time-dependent switch in MRI contrast from dark to bright with the release of Mn^2+^ from the NPs [62]. In order to dimmish levodopa’s side-effects, Cao and his-team used chitosan-coated levodopa liposomes to determine behavior and the effects of the expression of different molecules in levodopa-induced dyskinesia (LID)-induced model mice. Intravenously administration of chitosan-coated levodopa liposomes helped to reduce motor disturbances in PD mouse model compared to regular levodopa tablets [63]. For instance, Li and co-authors loaded levodopa into crystalsomes and demonstrated therapeutic efficacy in a PD mice model (Figure 4). Crystalsomes are nanoformulations synthesized from crystalized polymers that form crystalline structures under certain conditions [64]. The NPs demonstrated a particle size of 254 nm, a zeta potential of +6.87 mV, and an EE of 56%. An in vivo PD model mice pre-administered with two doses of levodopa crystalsomes showed ameliorated 1-methyl-4-phenyl-1,2,3,6-tetrahydropyridine (MPTP)-induced locomotor deficits, an enhanced expression of the tyrosine hydroxylase (TH) protein in striatal neurons, and an increased TH^+^ neuron expression in the substance nigra [65].

On the other hand, lipid-based NPs have been developed and demonstrated high biocompatibility and increased lipophilic or hydrophilic drug incorporation. One work demonstrated the incorporation of rasagiline in solid lipid NPs (SLNs) through a specific method of selection to ensure quickness, low waste, and low financial investment. An analytic hierarchy process was implemented, and rasagiline-loaded SLNs were prepared by a microemulsion technique that led to a yield of 83.6%. The average particle size was determined to be 248.7 nm, the zeta potential was around –35.5 mV, and the EE of 86.6% [66]. The further optimization of SLNs loaded with rasagiline was conducted using the microemulsion technique, and the effects of different formulations and processing parameters on physicochemical proprieties were evaluated [67]. In another study conducted by the same group, the in vitro drug release of SLNs loaded with rasagiline was analyzed. The results demonstrated a biphasic release pattern with initial 20% release over 30 min and then a sustained release of up to 98.2% over 24 h [68]. In addition, the stability analysis showed no aggregations or separation after six months in storage [69]. In order to intravenously administer SLN, a size smaller than 1 µm and sterilization are required when preparing new NP formulations to ensure high quality physicochemical characterization and a high stability. In addition, to ensure that the selection of the sterilization technique does not affect the developed nano-formulation, several techniques such as heat, filtration, and y-irradiation are available. For instance, SLNs loaded with rasagiline were subjected to moist heat sterilization, and the results demonstrated no changes in particle size, zeta potential, and drug encapsulation efficiency [70]. In the next article the authors showed the successful development of nanostructure lipid carriers (NLCs) as delivery systems. The main composition of the NLCs was acetyl palmitate (solid lipid), squalene (liquid lipid), Myverol (lipophilic emulsifier), and Pluronic F68 (PF68) (emulsifier). In addition, the authors added different additives such as forestall, T80, and distearoylphosphatidylethanolamine (DSPE)-PEG to the formulation in order to optimize the nanodelivery system to target the brain. A particle size analysis showed a mean diameter of 396 nm of the basic NLC. The incorporation of T80 resulted in an enhanced particle size of 431 nm, and the incorporation of DSPE-PEG showed a smaller size of 372 nm. The use of T80 and DSPE-PEG by the authors ensure higher brain drug accumulation, and the in vivo administration of these NLCs showed a more efficient delivery to the brain when compared to the aqueous solution [71].

When a pharmaceutical compound has insufficient stability or is unable to transgress the BBB and reach the brain, prodrugs can be designed to overcome these limitations. One example of a widely used prodrug in PD treatment is levodopa. In another article, the authors used previously-developed apomorphine prodrugs and encapsulated them into NLCs in order to improve solubility and achieve drug control release while avoiding the amount of apomorphine injections. The authors successfully demonstrated slower releases of apomorphine prodrugs of around 35% and 25% after 12 h. A hemolytic activity analysis demonstrated low levels of the hemolytic effect, and the lactate dehydrogenase (LDH) release from neutrophil was similar to the control. Additionally, both assays demonstrated that the prepared NLCs are biocompatible and safe. Moreover, the intravenously-administered NLCs loaded with apomorphine prodrugs were able to cross BBB and accumulate in the midbrain (which is one of the critical sites of action for PD) [72].

The use of liposomal formulations is gaining a lot of attention due to their non-toxicity and structural similarity to cells. Taking this in consideration, Hsu and his team prepared pegylated liposomes via the solvent spherule method followed by sonication in order to deliver apomorphine to the brain through parenteral formulation. The laser scattering data showed that the addition of T80 increased the mean particle size to 268.8 nm from the particle size of 150.9 nm from DSPE-PEG addition. Furthermore, the authors demonstrated that optimize liposomes had EE of 82.7% and 69.5% with the DSPE-PEG and Brij 78 formulation and the DSPE-PEG and T80 liposome formulation, respectively. The authors added Brij 78 or T80 because several studies have shown increase penetration with the use of these molecules. The in vivo administration of the DSPE-PEG and Brij 78 liposome formulations demonstrated a protective effect on drug stability and a steadier drug release in the brain compared to the free form [73]. In another article, Wen and his team decided to develop a multifunctional liposome with sustained imaging and the delivery of apomorphine as a model drug. The physicochemical characterization of the liposomes demonstrated a particle size of 133 nm and a zeta potential of +50 mV and EEs of 99% and 75% for quantum dots and apomorphine, respectively. The in vivo analysis demonstrated significant systemic distribution, increased brain uptake for the liposomal group when compared with control. Though liposome formulations exhibited stronger signals in the brain, there were systemic accumulations in other organs similar to what was observed in the free control. Regarding apomorphine delivery, the data showed a two-fold increase of brain uptake for the liposome formulation compared to the control [74].

Niosomes are similar to liposomes, but they are non-ionic surfactant vesicles and can be used as a good alternatives for BBB penetration. Gunay and his team formulated new pegylated liposomes and niosomes to encapsulate and target the brain delivery of pramipexole. Physicochemical data showed a similar particle size of around 100 nm, a zeta potential of −20 mV, and an EE of around 9%. In vivo rotational behavior test demonstrated that both liposome and niosome pegylated loaded with pramipexole had no differences between treatments when compared with table formulation. Even though the authors obtained similar results to that when using conventional dosage, it is still important to consider that a single dose of these nanodelivery delivery systems is nine-times smaller than that of pramipexole [75].

### 3.2. Transdermal Delivery

Several PD drugs have to be administered orally and present a low bioavailability after oral administration. Optimal pharmaceutical bioavailability is crucial to ensure sustained clinical response between individuals and minimize the development of motor fluctuations due to long-term therapy. The transdermal route could permit continued drug delivery, enhance patient compliance, and deliver directly to the circulation [76,77]. However, these systems are not yet fully developed, and it is a challenge for most drugs to be able to pass through stratum corneum of the skin. Many penetration enhancers such surfactants, iontophoresis, microneedles, and microwaves have been developed. However these strategies are associated with skin toxicity issues. In addition, transdermal drug therapy can have different absorption rates between individuals depending on the skin condition of the patient, so ensuring a decrease inter- and intra-individual variety is needed [77]. Currently, there are some transdermal patches available to treat PD and deliver rotigotine (Neupro^®^) [78] and selegiline (Emsam^®^) [79] to treat major depressive disorders. So, the use of nanoemulsions for transdermal permeation enhancement have demonstrated an increased percutaneous absorption of both hydrophilic and lipophilic drugs. The nanocarriers used for transdermal delivery usually include liposomes, niosomes, dendrimers, polymer NPs, lipid-based NPs, and nanoemulsions [80,81,82]. When developing a transdermal system, possible skin reactions such as skin rash, redness, and irritation must be considered. Some selegiline transdermal systems in clinical trials have demonstrated some side-effects in the skin of patients. As such, Fang and his team developed a novel transdermal system to deliver selegiline with a hydrogel-based formulation and a polyethylene microporous membrane (Solupor). The results demonstrated that the Solupor membrane was the rate-limiting compound, even with hydrogel present in the formulation [83]. Azeem and his-team proposed that the developed transdermal system could ensure the continuous delivery of ropinirole and prevent the onset of levodopa motor complications. For this, the authors used a previously-optimized nanoemulsion formulation composed of 5% Capryol 90, 25% Tween 20, and 0.75% (NPs with this formulation were called RPTNG). The results demonstrated a 7.5-fold increase in the skin permeation of ropinirole in vitro studies [84,85]. In addition, histopathologic studies demonstrated that RPTNG formulations were non-irritating to the skin, with no sign of skin edema or inflammatory cell infiltration. The pharmacokinetics analysis showed the better skin absorption of ropinirole from the nanoemulsion gel formulation when compared to tablets or conventional gel. RPTNG showed increases of ropinirole bioavailability of 2.26- and 7.69-fold compared to a conventional tablet and a reference ropinirole gel formulation, respectively. Moreover, in vivo studies in mouse model of PD showed an enhanced therapeutic effect from transdermal delivery compared to conventional tablets [86]. In another work, Chen and his-team developed a thermosensitive hydrogel to deliver selegiline through the transdermal route. The transdermal system was based on an alginate/Pluronic F127 (PF127) graft copolymer-prepared hydrogel. The results obtained from permeability studies demonstrated that selegiline permeation was more sustained with thermogels containing the alginate graft copolymer, and the release profiles showed that the alginate/PF127 hydrogel was best able to retain selegiline. The use of these type of nanoformulations and hydrogel has demonstrated reductions of these absorption discrepancies and enabled sustained drug release and constant drug absorption [87]. Moreover, Liu and his team used the diester prodrugs of apomorphine and developed a transdermal delivery system with nanosized lipid emulsions. The formulations were prepared with 12% mineral oil, 0.3% Myverol, 2.5% PF68, and 1.87 mM apomorphine. The permeation results demonstrated that application of the nanoemulsion increased diester prodrug permeation by around 11-fold compared to apomorphine HCl [88]. Patil and his team developed colloidal soft nanocarriers for transdermal ropinirole delivery. In this work, turbidimetric studies were performed to evaluate different surfactants on the emulsification process. The results demonstrated that T80 and T20 presented a good ability to emulsify Capryol™ 90 and Capmul^®^ glyceryl mono-dicaprylate (MCM), and a physicochemical analysis demonstrated a particle size of 160 nm and a zeta potential −4.24 mV. Permeability studies conducted on rat skin showed that the transdermal administration of nanoemulsions could increase permeation by 3.5-fold compared to hydrogel formulations. Furthermore, a ropinirole nanoemulsion demonstrated neuroprotective effects, improved locomotor activity, and reduced oxidative stress induced in a rotenone PD mice model [89]. Another work conducted by Sintov and his-team demonstrated the successful development of a self-assembling nanomicellar system that enabled the transdermal delivery of levodopa and carbidopa. The authors demonstrated the enhanced permeability accumulation of both drugs in blood analysis after 1 h of 0.6 µg/mL of levodopa and 0.45 µg/mL of carbidopa. These nanomicellar systems were able to efficiently co-deliver both pharmaceutic compounds to the systemic circulation [90]. In another study, selegiline was encapsulated in PLGA NPs and further added into ethylene vinyl acetate transdermal films. A physicochemical analysis demonstrated that the double emulsion solvent evaporation method produced NPs with a particle size 286.1 nm, a zeta potential of −29.11 mV, and a drug concentration of 97%. The authors demonstrated similar C-max values obtained from the intravenous administration of selegiline and the transdermal delivery of selegiline. In vivo brain distribution studies demonstrated an increase brain uptake of an intravenously administered selegiline solution, though with a short duration. However, the NP formulation showed a delayed drug brain uptake but a longer-lasting effect. Whereas, NPs formulation showed a sustained drug brain uptake and long duration effect. Furthermore, this strategy was able to decrease cataleptic response in mice, increase dopamine level, decrease MAO-B activity and skin test demonstrated safety for up to 24 h usage [91].

In another study, ropinirole was fully delivered through the transdermal route with the use of SLN and NLC systems. The in vivo transdermal administration on mice nude skin showed a 2.0 and 2.2-fold enhance of ropinirole bioavailability when compared with ropinirole oral solution and 1.8 and 1.2-fold increase when compared to oral bioavailability of SLN and NLC formulations. Pharmacokinetic studies that showed transdermal formulations were able to restore the biochemical changes of haloperidol PD model mice [92].

### 3.3. Intranasal Delivery

The BBB present a challenge for the efficient delivery of certain PD drugs. Intranasal administration is considered to be an alternative, non-invasive route that is able to circumvent the BBB and hepatic first metabolism passage. Intranasal administration can be delivered directly to the brain by olfactory or trigeminal nerve [93]. Several studies have demonstrated that the use of this type of administration allows for higher brain accumulation and less systemic exposure, which is crucial to decrease the side-effects of PD drugs. Still, drug nasal delivery by conventional gel formulations lacks some effectiveness. The use of nanoformulations in a formulation permits the more controlled release and prolonged duration of drugs, increase surface area, and decrease mucocilliary clearance [94,95]. For instance, optimized SLNs loaded with ropinirole were developed using factorial design (Design Expert^®^). The optimal formulation presented a particle size of 66.22 nm, a zeta potential of −2.819 mV, and an EE of 61.9%. Toxicity studies were conducted on sheep nasal mucosa to ensure SLN loaded ropinirole safety, and a pathologic analysis demonstrated no signs of severe of damage and no necrosis of epithelial cells. Release studies at pH 6.6 demonstrated sustained release with about 70% ropinirole release after 8 h. In addition, the authors demonstrated that NPs were stable for a period of three months and intranasal administration achieved a higher therapeutic activity in small dosages compared to a tablet dose formulation [96]. In the same year, the authors produced another type of NP to encapsulate ropinirole: an intranasal carrier made by a polymeric hybrid NP loaded with ropinirole. These new formulations presented better physicochemical characteristics, with a particle size of 98.43 nm, a zeta potential of 29 mV, and an enhanced EE of 72.7%. An ex vivo permeability analysis demonstrated a mucoadhesion of 78.76% and a diffusion of 61.34% of ropinirole through sheep mucosa. An in vivo treatment on a PD mice model demonstrated decreases in tremors, rigidity, and immobility compared to a commercial tablet formulation [97]. In the following years, Pardeshi and his team predicted molecular-level interactions and developed new self-assembling ropinirole nanocomplexes. The nanoplex form was able to release ropinirole in a controlled manner, and ex vivo permeation studies showed a permeation of 38.9% and a mucosa surface recovery of 52.3% [98]. Another work demonstrated the successful encapsulation of ropinirole in polymeric NPs. PLGA-TPGS-NPs were prepared by nano precipitation and presented sustained release for up to 14 h. Ex vivo permeation studies demonstrated an enhanced polymeric nanosystem drug permeation of 97.9% compared to 74.4% of a drug solution. A nasal cavity analysis demonstrated the integrity of the nasal mucosa after the administration of nanoparticulated loaded with ropinirole [99]. In a different work, Lungare and his team developed an intranasal spray for amantadine delivery with the use of thermo-responsive polymers and hydrogel-based formulations. The authors prepared 32 formulations, and only three were demonstrated to be optimal gelatin key determinants of residency within the nasal cavity [100].

Selegiline is an MAO-B inhibitor that presents some level of antioxidant activity in neurons. Kumar and his team developed a nanoemulsion to deliver selegiline through the nasal cavity and evaluated the antioxidant activity and therapeutic PD effect on a haloperidol-induced mice model. The results demonstrated that nanoemulsion administration inhibited free radial formation and presented antioxidant action. In addition to this, nasal administration showed a selegiline brain accumulation of 0.562 ng/mL, which was higher than the 0.175 ng/mL found after the intravenous administration of a free solution [101]. Salatin and his-team further explored the use of thermoreversible hydrogel for the intranasal delivery of selegiline. The results demonstrated that the optimal formulation had a 78.8% release of selegiline in around 480 min, which stable for up to three months and presented no damage to the nasal epithelium [102]. In another study, the same authors studied the therapeutic efficacy of these nanodelivery systems on 6-hydroxy dopamine model PD mice. A pharmacodynamic analysis demonstrated the efficient delivery of rotigotine to the brain, neuroprotective effects with the upregulation of TH expression, and reduced dopaminergic neural loss [103].

A different strategy was applied by Tan and his team in order to develop a new intranasal delivery system. For this, the authors prepared nanoformulations composed of diblock phenylboronic acid polycarbonate co-polymers containing a pH-responsive borate ester bond. The intranasal formulation demonstrated high loading levels of apomorphine and a pH-responsive apomorphine release at pH 5.5. In addition, the encapsulation of apomorphine conferred protection from oxidization after 60 min [104].

PLGA NPs were prepared to intranasally deliver levodopa. The in vivo results demonstrated different outcomes generated from the different types of administration: the effects of oral levodopa were prominent after 120 min, and intranasal levodopa relieved motor symptoms in the mice after 30 min. The concentration values obtained from the intranasal administration were highest in the brain and lowest in the systemic circulation [105].

Another study demonstrated that a formulation of NLCs of ropinirole could enable increased nasal administration. The authors found that the best formulation presented a particle size of 138 nm, a zeta potential of −20.15 mV, and an EE of 82.8% [106].

**Figure 5:**
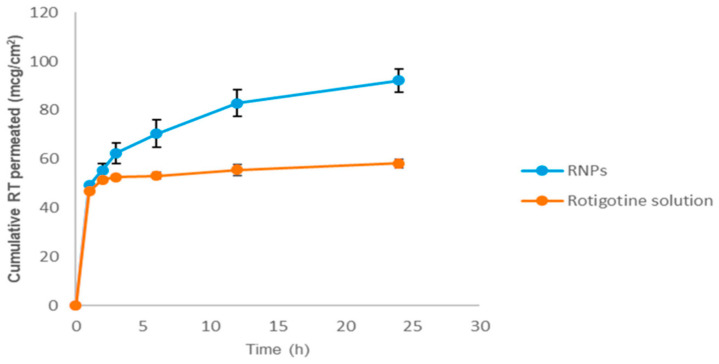
Ex vivo nasal permeation studies of rotigotine solution and rotigotine NPs in PBS at a pH of 7.4 (Reproduced from [112], MDPI, 2019).

Chitosan mucoadhesion proprieties can be retained in formulations and avoid rapid mucociliary clearance. Several studies have used chitosan NPs as intranasal delivery carriers due to their mucoadhesive proprieties, site-specific targeting, and controlled release nature. In this article, the authors developed chitosan NPs loaded with levodopa that were incorporated into thermo-responsive gel. The overall intranasal delivery demonstrated that thermo-responsive gel prepared with PF127 did not work in their favor and decreased the levodopa permeation uptake when compared to chitosan NPs alone. Still, the authors demonstrated a maximum drug recovery of 74.7% after nose-to-brain delivery [107]. For instance, Gulati and his team developed chitosan NPs to intranasally deliver selegiline and demonstrated an initial 4.38% drug release followed by a sustained release of 76.7% within the next 28 h [108]. Jafarieh and his team also prepared chitosan NPs to deliver ropinirole through ionic gelation. The optimized formulation demonstrated a particle size of 173.7 nm, a zeta potential of +32.7 mV, and an EE of 69.6%. Ex vivo nasal permeation was 76.1% and 35.67% ropinirole recovery rates for chitosan NPs and a free formulation, respectively. In vivo biodistribution studies demonstrated a higher accumulation of radio-labelled drugs in the brain after nasal administration [109]. In another work, a chitosan nanoemulsion loaded with ropinirole demonstrated therapeutic effects in a haloperidol-induced PD mice model, and intranasal administration allow increase brain accumulation and decrease blood accumulation [110]. Moreover, Raj and his-team used chitosan NPs for nose-to-brain delivery of pramipexole. Both in vitro and ex vivo goat nasal mucosa-permeating studies demonstrated higher permeation rates with the use of chitosan NPs to deliver pramipexole. The in vivo PD models with the use of both intranasal mucosa and chitosan NP demonstrated improvements in locomotor activity and restoration of dopamine levels up to 97.4 ng/g tissue when compared to the control group 137.32 ng/g or disease control group 48.33 ng/g [111]. In another work, chitosan NPs were developed to deliver selegiline. Pharmacodynamic studies on rotenone-induced PD mice demonstrated that intranasal administration improved the locomotor activity score, reduced the catalepsy score, and increased dopamine levels by two-fold compared to an oral formulation [112]. Another study conducted by Tzejung and his team successful produced chitosan NPs for intranasal rotigotine delivery. The optimal NP formulation presented a mean particle size of 75.37 nm, a zeta potential of +25.53 mV, an EE of 96.08%, and a mucoadhesion of 80.98%. As expected, the incorporation of rotigotine into chitosan NPs showed better cumulative drug permeation through the nasal mucosa (92.15%) than free solution (58.22%) (Figure 5). The safety of chitosan NPs was further proven with the maintenance of nasal integrity after mucosa administration [113].

After one year, the authors performed more tests to ensure the beneficial activity of chitosan NPs. The experiments conducted on the dopaminergic neuroblastoma cell line SH-SY5Y with the intranasal administration of chitosan NP-loaded rotigotine decrease synuclein alpha gene (SNCA) expression and increase superoxide dismutase enzyme levels to values close to those of the control. This data demonstrated that rotigotine induced antioxidant activity with a direct influence on an increase of radical scavenging enzymes. Furthermore, an in vivo pharmacokinetic analysis demonstrated an increased amount of rotigotine to the brain with C-max values of 61.72 ng/mL for intranasal delivery and only 36.92 and 5.35 ng/mL for intravenous and oral administration, respectively [114].

Nanoemulsions are good carrier delivery systems for the intranasal delivery of lipophilic drugs, and they provide large surface areas and protection from chemical and biological degradations. To ensure that nanoemulsions adhered to a mucosal membrane, Choudhury and his-team decided to coat them with chitosan molecules. The nanoemulsions were composed of Capryol 9, Tween 20, ethanol, and a polymer of chitosan; they were prepared by an aqueous titration method. The obtained results demonstrated the higher mucoadhesive strength of nanoemulsions coated with chitosan and a slow rate release profile after 8 h of incubation when compared to non-coated nanoemulsions. In addition, ex vivo permeability assays showed that coated nanoformulations had a higher steady flux of 3.97 µg/cm/h and a higher permeability coefficient 3.97 × 10^−3^ cm^2^/h compared to the values obtained for non-coated nanoformulations—2.82 µg/cm/h and 2.82 cm^2^/h, respectively [115].

### 3.4. Oral Delivery

One of the most common routes of drug administration is oral delivery, which provides a high level of patient compliance, is non-invasive, and is painless, even though high levels of bioavailability depend on drug solubility and permeability through the gastrointestinal tract. The use of nanotechnology has been widely explored to enhance gastric and intestinal adsorption, as well as to increase systemic bioavailability [116]. For instance, apomorphine is unable to be orally administered due to its low half-life, rapid degradation in the gastrointestinal tract, and first-pass effect, all of which contribute to a bioavailability of 1.7%. In order to facilitate oral bioavailability and brain-targeting, SLNs loaded with apomorphine were developed. A formulation optimization process was conducted and demonstrated that the formulated NPs were stable for up to 21 days in storage with a high entrapment efficiency of 90%. Stability studies of SLNs in the presence of a gastric medium and pancreatic enzymes showed increased and decreased particle sizes, respectively, though all SLN-formulated systems were able to protect apomorphine from degradation. Pharmacokinetic studies demonstrated that one dose of SLNs loaded with apomorphine had an absolute bioavailability of around 25%, whereas an oral solution only had an absolute bioavailability of 2.1%. In addition, the oral SLN formulation was able to reach the mice brains in the same quantities as in conventional treatment and promoted a higher therapeutic efficacy in 6-Hydroxydopamine hydrobromide (OHDA)-treated mice when compared to an apomorphine solution [117]. In addition, Borkar and his-team prepared a lipid-based drug delivery system to enable the oral delivery of apomorphine prodrugs diester. The in vivo oral administration of lipid-based formulations provided a higher concentration of apomorphine in the plasma when compared to an aqueous suspension. The prolonged release of apomorphine and gastrointestinal absorption enabled a constant plasma concentration within an optimal therapeutical window, thus resulting in side-effect reduction [118].

Another PD pharmaceutical compound with a significantly low oral bioavailable (less than 10%) and a short half-life is selegiline. In the attempt to increase selegiline’s oral bioavailability and potentiate its therapeutic action, Al-Dhubiab and his team prepared selegiline nanospheres. The physicochemical analysis of the NPs demonstrated an average particle size of 102 nm, an EE of 98.4% and the controlled release of selegiline. Still, further studies are needed to ensure feasibility for oral delivery [119].

Papadimitriou and his team developed chitosan NPs loaded with pramipexole in order to reduce daily intake and enable controlled drug release. Through the ionic gelatin method, chitosan NPs were prepared and showed an average particle size of 289 nm, a zeta potential of −29.17, and an EE of 18.9%. In vitro release studies simulating a gastric environment demonstrated a pramipexole release of 100% within 6 h and a steadier release a simulated intestinal environment of around 50.8% within 1 h [120]. Another study demonstrated the safety of newly hollow mesoporous silica (HMS) NPs and the ability to efficiently incorporate pramipexole. In vitro drug release studies have demonstrated a sustained release pattern at both intestinal and gastric pH values. In addition, no hemolysis was found for HMS-pramipexole NPs, and SH-SY5Y treatment resulted in a better antioxidant protection effect when compared with pramipexole alone [121]. In the same year, Tzankov and his team developed another type of mesoporous silica NP (MCM-41) to incorporate into pramipexole for oral administration. This strategy proposed the use of pH-dependent polymer coated with sodium alginate (protector during stomach passage) and chitosan (enabled the transport of polar drugs in epithelia surface). The average particle size was 420–570 nm, and the average zeta potential was –40 mV. No toxicity was observed for up to 42.24 µm, and there was around 15–20% neuronal protection against H_2_O_2_ [122].

Another strategy that can be used for efficient oral delivery is the development of gastroretentive drug delivery systems. These systems allow for the enhancement of gastric residence time, which results in an improve availability of a drug to its adsorption site. Several mechanisms can be explored to ensure gastric retention such as high density, gastric adhesion, swelling, and floating. Ngwuluka and co-authors decided to combine more than one mechanism and nanoparticle encapsulation to enhance levodopa’s pharmacokinetic profile. The overall results exhibited a constant delivery of levodopa over a longer period of time than in conventional dosage forms [123].

### 3.5. Buccal Delivery

**Figure 6:**
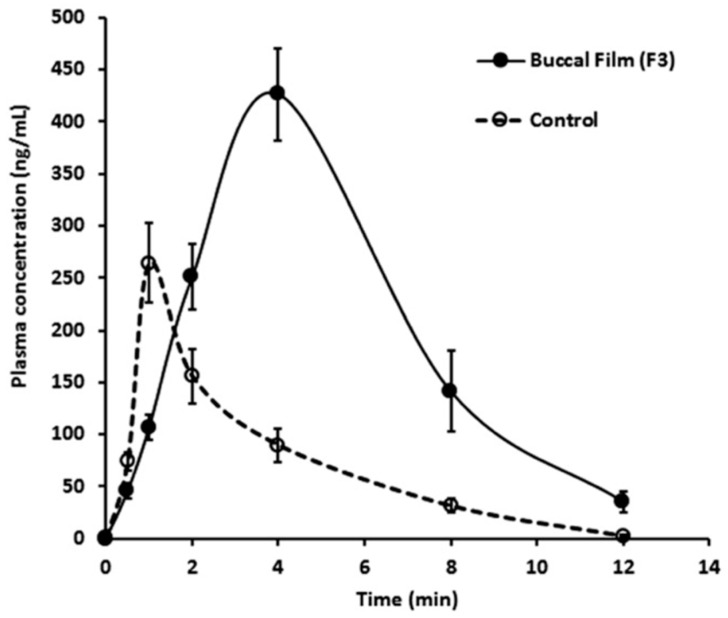
Comparison of the plasma profiles of selegiline following the buccal application (4 h) of nanosphere-loaded buccal film and control solution via the oral route in rabbits (Reproduced from [127], Taylor & Francis, 2014).

The conventional oral therapy of several current PD drugs has shown weak pharmacokinetic profiles, with a low bioavailability and extensive hepatic metabolism. Dysphagia is characterized by an inability to swallow and is a common symptom presented by PD patients that limits oral drug administration [124]. Other types of alternative routes have been explored in order to more efficiently deliver PD compounds. The buccal route can directly deliver a drug into systemic circulation, avoiding gastrointestinal degradation and bypass first hepatic metabolism. In addition, the buccal area can reach the area of 50 cm^2^ for drug permeation. Nevertheless, efficient drug delivery through buccal mucosa has several disadvantages such as a low drug permeability and a low drug residence time. To overcome these limitations, the use of NPs can be a useful approach to increase drug permeation and expand residence time [125,126]. The work of Al-Dhubiab and his team used already-prepared PLGA NPs loaded with selegiline and embedded them into the buccal films. Several analyses—such as for pH, thickness, bioadhesion, selegiline release, amount of drug, swelling capacity, mucoadhesive strength, and permeation—were conducted by the authors to ensure the safety and efficacy of the buccal films. The overall results demonstrated a good mucoadhesion in rabbit buccal mucosa, a significantly higher drug permeation with a steady-state flux of 70.25 mg/cm^2^/h, a prolonged absorption, and 1.6-fold higher C-max values than those of the control (Figure 6) [127].

For instance, apomorphine is able to passive diffuse rather that active transport. In this article, the authors chose apomorphine as a PD model drug to enable steady-release buccal administration to substitute current parenteral administration with subcutaneous infusion. For this, the authors compared different types of buccal enhancer permeability solutions (organic solvents) to a nano-liposphere formulation. The ex vivo permeability assays demonstrated that use of organic solvents produced significant increase on apomorphine permeation. When administered with nano-liposphere formulation, the apomorphine permeation lag-time increased to up to 3 h, compared to the 1 h of the control [128].

## 4. Nanotheranostics in PD

Developing therapeutic and diagnostic tools to target the brain is a challenge due to the presence of the protective BBB. These mechanisms are crucial to ensure brain homeostasis and protection against toxins, pathogens, and inflammation. The use of nanotechnology allows for the bypass of the BBB, surface functionalization to the target, penetration of the brain, and increased pharmacokinetic profiles. Thus, nanotheranostics can be an excellent future alternative to embrace the use of nanoparticles and construct a multifunctional system that is able to diagnose, monitor, and deliver therapeutic compounds to the brain. Several studies have demonstrated the successful use of those strategies in cases like cancer, atherosclerosis, and gene delivery [129]. However, few studies have so far been conducted with the use of this strategy in neurodegenerative diseases. For instance, the early diagnosis and monitoring of PD progression are hard to achieve, and different techniques have been explored to overcome these clinical limitations [130]. The successful early diagnosis of PD can be achieved via the determination of changes in the molecular level before PD symptoms appear, but initial molecular targeting is still lacking. For now, D2 and D3 receptors are widely used to control and reach a PD diagnosis with the use of single-photon emission computed tomography (SPECT) imaging [131]. Thus, the development of new specific imaging and biolabeling ligands is needed to improve early diagnosis. The use of biomarkers in the preclinical stage can be useful to identify high-risk patients and treat them before motor symptoms appear. Still, detection in blood can be difficult due to low numbers of molecular targets underlying early PD, so the development of more sensitive detection techniques is needed. Several NP strategies have been developed to target and quantify α-syn [132,133], dopamine levels, and mitochondrial dysfunction [134,135]. Yang and his team determined α-syn blood concentration with the use of an immunomagnetic reduction assay and the utilization of antibody-functionalized magnetic NPs. The results demonstrated that the assay was sensitive and able to detect α-syn in the blood and present differences levels between PD patients and healthy patients [136]. Another type of assay was used to quantify α-syn oligomers. The use of a fluorescence intensity distribution analysis assay was explored and demonstrated to be successful for α-syn oligomers quantification [137]. More studies have been conducted in order to determine the feasibility of α-syn determination for biomarking through blood analysis [138], cerebrospinal fluid analysis [139,140], and non-invasive brain detection through MRI analysis [141]. For instance, Toshi and his team developed a quantum dot composed of an L-lysin FeO_2_ NPs system that was able to track and inhibit α-syn aggregation in live cells [142]. Moreover, Malvindi and his team designed a quantum dot nanorod for both imaging and dopamine delivery with the ability to cross an in vitro BBB membrane with GLUT-1 transporters [143]. Another type of NP was used as theragnostic system with the use of dextran supermagnetic iron oxide (SPIONS) to label and track transplanted cells. The authors demonstrated the successful MRI tracking analysis of cells in vivo. The results demonstrated that the presence of SPIONS did not affect cell cycle progression or differentiation capability, and they also demonstrated a tracking ability for at least six months [144]. In article, the authors used MRI-guided, ultrasound-delivered nano/microbubbles to transfect neurons with nuclear factor E2-related factor 2 (Nrf-2) and examined the effect of Nfr-2 overexpression. The results demonstrated neuroprotection agonist oxidative stress in dopaminergic neurons [145].

## 5. Conclusions and Future Perspectives

The treatment of PD is a controversial subject, in part due to the fact that there is not yet a defined diagnostic tool to detect the early pathologic pathway and neurodegeneration. Currently, pharmacologic therapy starts when the patient presents motor symptoms. However, when motor symptoms appear, around 50–70% of dopaminergic neurons have already been lost [146]. This poses a huge challenge for therapeutic compounds to present higher brain effectiveness and delay disease progression. Moreover, there is not a direct link that assumes that early treatment will present differences on the impact of disease progression. Current therapeutical strategies are mainly focused on slowing down and reversing motor symptoms. This type of treatment is able to provide an expansion of the quality of a patient’s life, but chronic treatments start loses effectiveness and increase side-effects overtime. Several studies presented in this review have demonstrated that the use of nanosystems could potentiate sustained release, decrease side-effects, decrease dosage, and increase the effectiveness of repurposed PD drugs. Moreover, the use of alternative routes of administration can be crucial to enhance patient complacency, facilitate drug administration in later stages of the disease, lowering drug dosage and possibility to circumvent metabolic pathways and BBB that limited drug full efficacy. Still, for PD, there is currently no a disease-modifying therapy that could allow for complete and full neuroprotection. Massive efforts have been made to develop new effective PD therapeutics that include newly created compounds to decrease motor fluctuations (e.g., safinamide and opicapone) [147,148,149,150], α-syn target therapies [151], brain insulin pathways, and experimental therapies (stem neuronal cell transplantation, bright light therapy, and gene therapy) [152]. Overall, nanoparticles can be considered to comprise useful strategy for drug repurposing against PD. Nanotheranostics is a still-growing area for the CNS approach and, in the future, could be a huge strategy for efficient clinical diagnosis and improvements of directional and efficient treatment.

## Data Availability

Not Applicable.

## Abbreviations

AADC—Aromatic L-amino acid decarboxylase; BBB—Blood brain barrier; CNS—central nervous system; COMT—Catechol o-methyltransferase; EE—encapsulation efficiency; DOPAC—dihydroxyphenyl acetic acid; HMS—Hollow mesoporous silica; DSPE—distearoylphosphatidylethanolamine; LDH—lactate dehydrogenase; LID—Levodopa-induced dyskinesia; MAO-B—mono-amide oxidase B; MCM—glyceryl mono-dicaprylate; MPTP—1-methyl-4-phenyl-1,2,3,6-tetrahydropyridine; MRI—Magnetic resonance imaging; NLC—Nanostructure lipid carriers; NMDA—N-methyl-D-aspartate; NPs—Nanoparticles; Nrf-2—Nuclear factor E2 related factor 2; OHDA—6-Hydroxydopamine hydrobromide; PD—Parkinson Disease; PEG—polyethylene glycol; PET—Positron Emission Tomography; PF127—Pluronic F127; PF68—Pluronic F68; PHBV—Poly-(3-hydroxybutyrate-co-3-hydroxyvalerate); PLGA—Poly lactic-co-glycolic acid; PLLA—Poly (L-lactic acid); PVA—Poly (vinyl alcohol); MTPT—1-methyl-4-phenyl-1,2,3,6-tetra-hydropyridine; SLN—Solid lipid nanoparticles; SNCA—Synuclein alpha gene; SPECT—single-photon emission computed tomography; SPIONS—Supermagnetic iron oxide NPs; T80—Polysorbate 80; TH—Tyrosine hydroxylase; UPS—Ubiquitin proteosome system.
